# Synthetic computed tomography techniques for adaptive proton therapy in head and neck cancers^☆^

**DOI:** 10.1016/j.phro.2025.100847

**Published:** 2025-10-09

**Authors:** Suryakant Kaushik, Nadine Vatterodt, Jakob Ödén, Albin Fredriksson, Stine S. Korreman, Iuliana Toma-Dasu

**Affiliations:** a RaySearch Laboratories AB (Publ), Stockholm, Sweden; b Department of Physics, Medical Radiation Physics, Stockholm University, Stockholm, Sweden; c Department of Oncology and Pathology, Medical Radiation Physics, Karolinska Institutet, Stockholm, Sweden; d Department of Clinical Medicine, Aarhus University, Aarhus, Denmark; e Danish Center for Particle Therapy, Aarhus University Hospital, Aarhus, Denmark

**Keywords:** Synthetic CT, CBCT image correction, Adaptive proton therapy, Head and neck cancer

## Abstract

Head and neck (HN) radiotherapy often requires corrective interventions. This study evaluated three methods for synthetic computed tomography (CT) generation for adaptive HN planning using cone-beam CT (CBCT) images. CBCT images for 15 patients were paired with same-day repeat CT scans and robustly optimized proton plans were recalculated. The anatomy-preserving virtual CT (APvCT) method utilized organs-at-risk as deformation controlling structures. APvCT and conventional virtual CT methods showed lower mean absolute errors in CT number values compared to corrected CBCT; however, all synthetic CT methods were found suitable for proton dose recalculation with gamma passing rates greater than 96.7% (2%,2mm).

## Highlights

• Anatomy-preserving virtual computed tomography suitable for head and neck patients.
• Corrected cone beam and virtual computed tomography led to similar results.
• Mean absolute error ≤ 42 HU in all methods compared to repeat computed tomography.
• Gamma passing rate (2%, 2 mm) ≥ 96.7% for proton dose recalculation in all methods.

## Graphical abstract

**Figure:**
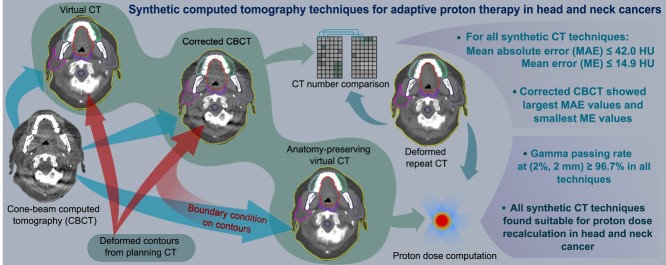

## 1 Introduction

Modern treatment planning techniques for radiotherapy, such as intensity modulated proton therapy (IMPT), utilize a complex arrangement of proton beam spots and intensities to achieve a highly conformal dose distribution. This helps reduce the dose to healthy tissues surrounding the tumor and has demonstrated potential advantages in head and neck (HN) cancer [1], [2]. However, different treatment sites can exhibit different anatomical variations [3], presenting particular difficulties in maintaining the quality of treatment. In the case of HN region, anatomical changes during the course of IMPT often require corrective interventions [4], [5], [6]. Implementing an adaptive treatment strategy to deal with daily anatomical changes that uses cone beam computed tomography (CBCT) to generate synthetic computed tomography (sCT) images can effectively maintain the quality of treatment [7]. Previous studies have indicated that deformable image registration (DIR) and projection-based methods are suitable for sCT generation and achieve high accuracy in proton dose calculations [8], [9]. Recent studies assessed the dose recalculation accuracy using two sCT techniques in a commercial treatment planning system (TPS), recommending the use of corrected CBCT (corrCBCT) over virtual CT (vCT) for the HN region [10], [11]. Although vCT has a CT-like image quality, it may not preserve daily anatomy. These studies also highlighted that corrCBCT depends on CBCT image quality with CT number accuracy decreasing as artifacts in the CBCT images increase. In another recent study, an anatomy-preserving virtual CT (APvCT) was proposed as an alternative sCT technique for use in online adaptive proton therapy, which demonstrated that this technique can maintain CT-like image quality and simultaneously preserve daily anatomical details under larger anatomical changes in patients with prostate cancer [12]. However, given that the anatomical changes in the HN region are not on the same scale as those observed in the prostate, the applicability of APvCT to the HN region might face different challenges.

This study aimed to implement the APvCT technique for the HN region and determined the potential benefits or drawbacks over the other two techniques, namely corrCBCT and vCT, by examining image quality metrics and dose recalculations.

## 2 Material and methods

### 2.1 Clinical data and treatment planning

This study retrospectively included anonymized data (data usage was approved by an internal review board) from 15 patients who received proton therapy for oropharyngeal cancer using the Varian ProBeam proton therapy system (Varian Medical Systems, Inc., Palo Alto, CA, USA) at the Danish Centre for Particle Therapy, Denmark. A planning CT (pCT) and 4–5 repeat CTs (reCTs), acquired on the same CT scanner, were available for each patient. CBCTs acquired during treatment positioning with an integrated imaging setup in the Varian ProBeam with available same-day reCTs were included, with a total of 100 CBCT/reCT pairs. Each pCT contained contours drawn by the radiation oncologist for targets and organs at risk (OARs).

The study was performed in a research version of RayStation 12 A (RaySearch Laboratories AB, Stockholm, Sweden); however, all functionalities are available in later commercial versions of RayStation. The nominal IMPT treatment plans were optimized on the pCT, using a standard machine learning-based IMPT planning model ‘RSL-IMPT-Oropharynx-7000-SIB (3.0)’ incorporating simultaneous integrated boost. These plans comprised five fields: left and right anterior obliques, left and right posterior obliques, and an additional lateral beam depending upon the tumor location. The posterior oblique beams and the additional lateral beam did not pass through the shoulders and the thorax region (inferior post neck region). The dose calculation was performed using a Monte Carlo algorithm (Dose Engine: Proton PBS Monte Carlo 5.4) in RayStation with a statistical uncertainty threshold of 0.5% and a constant relative biological effectiveness (RBE) value of 1.1. Clinical target volumes for primary and secondary targets (CTVp/CTVs) were prescribed 70.0 Gy (RBE)^1^ and 54.25 Gy (RBE), respectively, delivered in 35 fractions. Both targets were robustly optimized with ±3% density uncertainties and ±3mm setup uncertainties in all three translational directions.

### 2.2 Synthetic CT generation

Python scripts were developed within the RayStation scripting environment to limit human intervention in the results. Initially, a density table was assigned to all images, and an external region of interest (ROI) was auto-segmented on CT and CBCT images with a threshold value of −250 and −900 Hounsfield Units (HU), respectively. To improve DIR results for CBCTs with limited field-of-view (FOV), a FOV ROI was created using a TPS module and a cFOV ROI was generated by contracting the FOV with a uniform 2 cm margin as recommended by the vendor in this RayStation version. Initial frame of reference registrations were performed with pCTs as primary images and reCTs/CTs as secondary images. Three methods were used to generate sCT images as described below using standard RayStation functionalities [11], [12]:

A **Corrected CBCT (corrCBCT)** method utilizes an iterative algorithm for CBCT image correction that converts the CBCT values to the CT number values [10]. A DIR was generated between pCT and CBCT with cFOV as the focus ROI and using the correlation coefficient as a similarity measure within the RayStation DIR algorithm ANACONDA [13]. When the FOV in the CBCT was limited, corrCBCT did not contain the information outside the FOV, thus necessitating an FOV as an extra parameter to automatically copy the pixel values outside the FOV from the deformed pCT.

A **Virtual CT (vCT)** method deforms the pCT to the anatomy of the CBCT and applies low-density corrections by replacing mismatched low-density tissue regions, such as air cavities, with pixel values from the corrCBCT [10]. The same DIR from corrCBCT was used to generate the virtual CT.

An **Anatomy-Preserving Virtual CT (APvCT)** technique generates a vCT using a contour-driven DIR, where the DIR was created based on ROIs contoured on the corrCBCT and those present on the pCT [12]. These ROIs were called controlling ROIs and included the following OARs in this study: parotid glands, submandibular glands, oral cavity, mandible, buccal mucosa, thyroid, larynx, pharyngeal constrictor muscles, esophagus, and spinal cord. To generate the controlling ROIs, a DIR was created between pCT and corrCBCT using mutual information as a similarity measure, which was assessed to result in good anatomical matches between ROIs for these cases. Subsequently, these controlling ROIs were mapped to the CBCT from the corrCBCT using rigid registration. However, other methods to generate ROIs to be used as controlling ROIs could also be used. Finally, a contour-driven DIR was created between the pCT and CBCT images by applying boundary conditions on the controlling ROIs and cFOV as the focus ROI, using the correlation coefficient as a similarity measure. This new contour-driven DIR was used to generate a vCT.

### 2.3 Synthetic CT evaluation

The weekly reCT was deformed to the corresponding CBCT anatomy for each patient, resulting in a deformed reCT (def-reCT), which served as a reference image for comparison of the sCT image. In addition, a ROI analysis box was created on the pCT, which covers both the lateral and anterior-posterior extents of the HN region, as well as the superior-inferior extent of the targets. This analysis box was rigidly mapped to all def-reCTs and sCTs to define the region of importance and was used to limit evaluations within this box for our study.

The mean error (ME) and mean absolute error (MAE) with standard deviation (SD) were determined by pixel-by-pixel comparison of the CT numbers between def-reCT and sCT. For each type of sCT, the final ME and MAE values per patient were calculated as the average of all sCT images of that specific type.

The dosimetric assessment involved recalculating the nominal IMPT plan on all def-reCTs and sCTs for every patient. A three-dimensional global gamma index [14] was determined between the recalculated doses on def-reCT and their respective sCT, using criteria of (2%,2mm) and (1%,1mm), with a dose threshold of 10%. Subsequently, a gamma passing rate (GPR) was derived from this 3D global gamma index.

## 3 Results

**Fig. 1:**
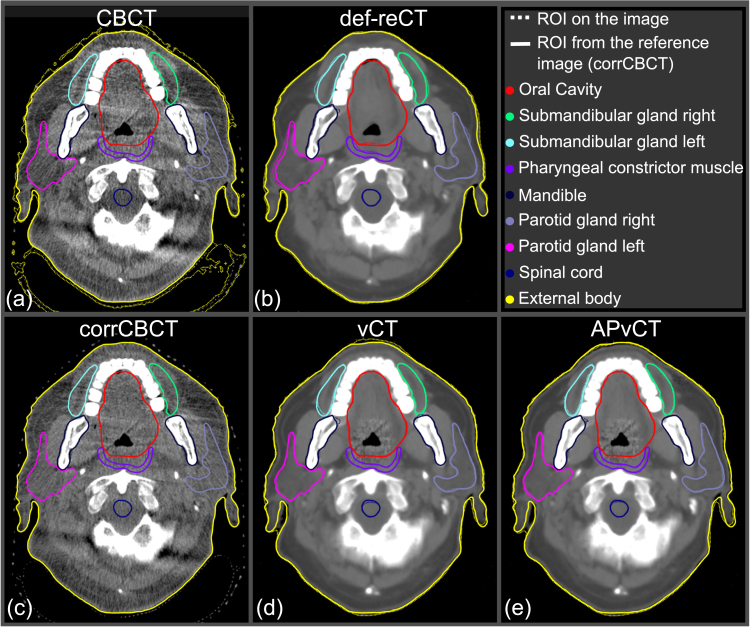
An axial CT slice from (a) cone beam computed tomography (CBCT), (b) deformed repeat CT (def-reCT), (c) corrected CBCT (corrCBCT), (d) virtual CT (vCT), and (e) anatomy-preserving virtual CT (APvCT). Separate deformable image registrations with mutual information as a similarity measure were created between planning CT and synthetic CTs/def-reCT to map the regions of interest (ROIs) to the image sets (with dotted lines). ROIs with solid lines denote the contours of reference daily anatomy from the corrCBCT.

Fig. 1 presents representative CT slices from CBCT, def-reCT, and the sCTs, all adjusted to a similar window level. Upon visual examination, the anatomical differences between the sCTs were minimal. However, the corrCBCT images appear grainier than those of vCT and APvCT.

**Fig. 2:**
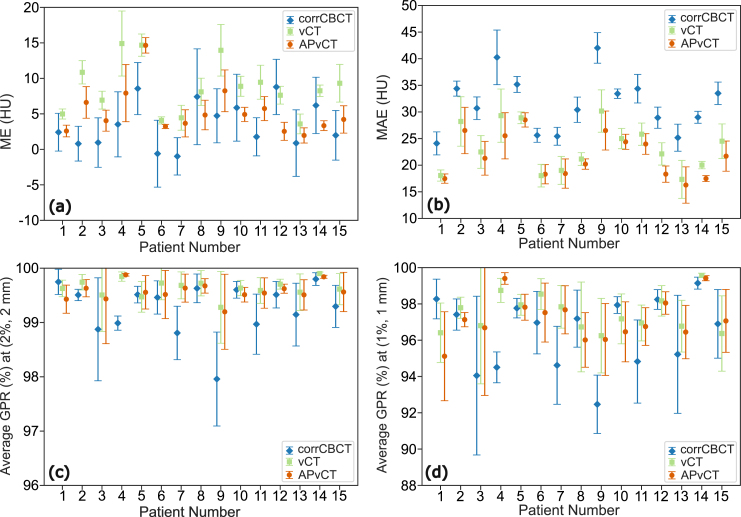
For each patient, (a) mean error (ME), (b) mean absolute error (MAE), average gamma passing rates (GPR) with (c) (2%,2mm), and (d) (1%,1mm) criteria of anatomy-preserving virtual CT (APvCT), virtual CT (vCT) and corrected cone beam computed tomography (corrCBCT) with respect to deformed repeat CT. The error bars represents one standard deviation.

The highest ME values (across all patients and image sets) were 8.8, 14.9, and 14.7 HU for corrCBCT, vCT, and APvCT, respectively, while the highest MAE values observed were 42.0, 30.2, and 28.5 HU. The average ME value (Fig. 2) among all patients, along with the 95% confidence interval, was 3.5 [1.7,5.3], 8.7 [6.6,10.7] and 5.2 [3.5,7.0] HU for corrCBCT, vCT, and APvCT, respectively. In contrast, for MAE, the average values were 31.5 [28.5,34.5], 23.3 [20.9,25.8] and 21.7 [19.5,23.9] HU.

The mean GPR (and minimum) values (Fig. 2) for corrCBCT, vCT and APvCT were 99.2% (96.7%), 99.6% (97.8%) and 99.6% (97.2%), respectively, under the criteria of (2%, 2mm). Under the (1%, 1mm) criteria, the values were 96.2% (86.3%), 97.4% (88.3%), and 97.1% (86.4%), respectively.

## 4 Discussion

This study presented the first application of the APvCT technique for the generation of synthetic images in patients with oropharyngeal cancer. The initial results demonstrated that APvCT is practical for generating synthetic images from daily CBCTs in a commercial TPS and can be utilized for dose calculations of IMPT treatment plans.

Among various sCT techniques, corrCBCT had the lowest ME values and the highest MAE values. However, the ME values had large standard deviations, which could be due to higher noise levels associated with corrCBCT. The study by Chang et al. [10] reported that corrCBCT exhibits higher GPR values compared to vCT and recommends the adoption of corrCBCT for HN patients, with a similar recommendation by Yeap et al. [11]. The recommendation was due to the dependence of the vCT on the DIR. However, an APvCT employs controlling ROIs to maintain anatomical integrity, which has the potential to decrease DIR errors. Our study found marginally higher GPR values for both APvCT and vCT compared to corrCBCT. This may be attributed to the reliance of corrCBCT on the quality of the initial CBCT images. Nevertheless, the lowest GPR value across all sCT techniques with (2%, 2mm) criteria was recorded at 96.7%, aligning with findings from other studies spanning 96.0% [9] up to 97.0% [10]. Thus, all sCT methods seem suitable for proton dose calculation and daily dose monitoring, consistent with the study by Vestergaard et al. [15]. As such, APvCT presents itself as a viable alternative to other sCT methods, which may be beneficial in scenarios where the CBCT images have significant artifacts or where there are significant anatomical changes.

The processing times for both corrCBCT and vCT have been investigated in an earlier study [10]. Compared to vCT, APvCT necessitates two additional steps: contouring on the corrCBCT and mapping of contours to the CBCT image. However, it does not extend the overall time in replanning workflows such as online or offline adaptive replanning, since plan adaptation requires targets and OAR contours, and contour mapping is extremely fast.

This study demonstrated that robustly optimized proton treatment plans recalculated on different sCTs yield comparable GPR results. Although 100 CBCT/reCT pairs were used to generate and assess sCTs, a limitation of this study was the relatively small cohort of 15 patients, and an increase in the number of patients could bring greater inter-patient variability. An additional limitation was the uncertainty in controlling ROIs generated with the auto-contouring method, which the APvCT technique, as it was used in this study, relies upon. An improved contouring method is likely to improve the APvCT images. However, it should be noted that contour uncertainty can lead to differences in dose-volume histogram metrics and affect patient outcome prediction, including tumor control probability, normal tissue complication probability, and overall survival [16]. Furthermore, if auto-contoured targets are not manually corrected or delineated, it can substantially affect the quality of adaptive plans in HN cancer [17]. Additionally, the study did not investigate the differences in treatment replanning using various sCT methods.

In conclusion, APvCT and vCT had lower MAE values and higher ME values compared to corrCBCT; however, the difference in gamma passing rate for dose recalculation of robustly optimized IMPT treatment plans was minimal. Therefore, for daily dose monitoring and to determine the necessity of adaptive treatment planning in HN cancer, we found all methods to perform adequately. However, the decision to apply any sCT technique should be based on the quality of the CBCT images and the extent of anatomical deformations. The contours mapped to the corrCBCT in this study are good enough for APvCT image generation and dose recalculation accuracy, but their accuracy for dose statistics evaluation or replanning remains to be investigated.

## CRediT authorship contribution statement

**Suryakant Kaushik:** Conceptualization, Methodology, Software, Validation, Formal analysis, Investigation, Data curation, Writing – original draft, Writing – review & editing, Visualization. **Nadine Vatterodt:** Methodology, Validation, Formal analysis, Resources, Data curation, Writing – review & editing, Visualization. **Jakob Ödén:** Conceptualization, Methodology, Writing – review & editing, Supervision. **Albin Fredriksson:** Conceptualization, Methodology, Writing – review & editing, Supervision, Project administration. **Stine S. Korreman:** Resources, Writing – review & editing, Project administration. **Iuliana Toma-Dasu:** Conceptualization, Methodology, Writing – review & editing, Supervision.

## Declaration of competing interest

The authors declare the following financial interests/personal relationships which may be considered as potential competing interests: Suryakant Kaushik, Jakob Ödén and Albin Fredriksson are employees of RaySearch Laboratories AB, Stockholm, Sweden. There are no other conflicts of interests to declare from all authors.

## Footnotes

1 Gy (RBE) refers to nomenclature of International Commission on Radiation Units and Measurements (ICRU) Report 93: Prescribing, Recording, and Reporting Light Ion Beam Therapy.
